# Role of animal models in biomedical research: a review

**DOI:** 10.1186/s42826-022-00128-1

**Published:** 2022-07-01

**Authors:** P. Mukherjee, S. Roy, D. Ghosh, S. K. Nandi

**Affiliations:** 1 Department of Veterinary Clinical Complex, West Bengal University of Animal and Fishery Sciences, Mohanpur, Nadia, India; 2 Department of Veterinary Surgery and Radiology, West Bengal University of Animal and Fishery Sciences, Kolkata, India

**Keywords:** Animal models, Ethical concern, Animal research, Human diseases, Biomedical research

## Abstract

The animal model deals with the species other than the human, as it can imitate the disease progression, its’ diagnosis as well as a treatment similar to human. Discovery of a drug and/or component, equipment, their toxicological studies, dose, side effects are in vivo studied for future use in humans considering its’ ethical issues. Here lies the importance of the animal model for its enormous use in biomedical research. Animal models have many facets that mimic various disease conditions in humans like systemic autoimmune diseases, rheumatoid arthritis, epilepsy, Alzheimer’s disease, cardiovascular diseases, Atherosclerosis, diabetes, etc., and many more. Besides, the model has tremendous importance in drug development, development of medical devices, tissue engineering, wound healing, and bone and cartilage regeneration studies, as a model in vascular surgeries as well as the model for vertebral disc regeneration surgery. Though, all the models have some advantages as well as challenges, but, present review has emphasized the importance of various small and large animal models in pharmaceutical drug development, transgenic animal models, models for medical device developments, studies for various human diseases, bone and cartilage regeneration model, diabetic and burn wound model as well as surgical models like vascular surgeries and surgeries for intervertebral disc degeneration considering all the ethical issues of that specific animal model. Despite, the process of using the animal model has facilitated researchers to carry out the researches that would have been impossible to accomplish in human considering the ethical prohibitions.

## Background

The animals used in various studies and investigations are related to the evolution of human history. Though there are many shreds of evidence that Aristotle in ancient Greece successfully used animals in understanding the human body, the main breakthrough in animal models happened in the eighteenth and nineteenth centuries with the scientists like Jean Baptiste Van Helmont, Francesco Redi, John Needham, Lazzaro Spallanzani, Lavoisier and Pasteur who studied the origin of life using animal models [1]. At the same time, human physiology, anatomy, pathology as well as pharmacology were also studied using animal models. With the remarkable advancements in drug development, biomedicine and pre-clinical trials, the importance of animal models has increased many folds in the last decades, as the therapeutic outcome and drug safety are the foremost important criteria for a drug and medical device considered to be used in the human model [2]. The scientific apply of animal models in the arena of biological research and drug development is an age-old practice because of the notable resemblance in physiology and anatomy between humans and animals, especially mammals [3]. One must consider that the physiological processes of humans, as well as mammals, are complex in terms of circulatory factors, hormones, cellular structures, and tissue systems. Hence, investigation of various aspects such as molecular structures, cellular and organ functions in physiological and pathological conditions must be taken into consideration.

The process of selection of an animal model for biomedical research is a very intricate part, as all models are not acceptable due to various limitations. Many factors should be taken into consideration during the selection of an ideal animal model for biomedical trials. The most important criteria are the proper selection of models in terms of resemblance between animal species and humans in terms of physiological and/or pathophysiological aspects. Detailed evaluation during the application of certain drugs/molecules/devices and their capacity to reproduce the disease or pathology at the same level as that of humans. Availability and the size of animal species under consideration. Long life duration of the animal species under study. A Large animal population in a model facilitates the availability of multiple sub-species.

Many animal species such as *Drosophila* (insects), *Danio rerio*, or zebrafish (fish), *Caenorhabditis elegans* (nematodes), *Xenopus* (frogs), and mammals such as mice, rabbits, rats, cats, dogs, pigs, and monkeys have been accepted worldwide for their phylogenetic resemblance to humans [4].

Choice of an appropriate animal model is most of the time a tedious job and sometimes depends on assumptions and convenience of the study and researchers without considering whether the model will be appropriate or not. Irrational selection of an inappropriate animal model for scientific investigations will yield incorrect findings, as well as fetch misusage of resources and lives. Moreover, it results in erroneous, duplicative, and inappropriate experiments [5]. To minimize these problems, recently researchers have advanced their researches to produce animal models that are very specific to the research under consideration. They produced custom-made transgenic animal models by incorporating genetic information directly into the embryo either by injecting foreign DNA or through retroviral vectors [6]. Through the incorporation of human cells into the recipient animals, researchers can study the effects of pathogens similar to the way in the human body [7]. Proper selection of animal models is mainly related to the nature of the drug or medical devices under study. In many instances, a single animal model is not able to signify a human disease alone, in that case, the combination of several models can potentially signify the procedure [8].

## Main text

### The significance and challenges of animals in biomedical research

There has always been a debate among the researchers about the significance of animal models, as many experiments yield promising results, whereas, others couldn’t produce desired outcomes, so, that model could be translated to humans too. Owing to their close phylogenetic closeness to humans, non-human primates are proved to be the most potential candidate. They have genetic, biochemical, and psychological activities similar to humans. In this context, the necessity of non-human primates continues to grow in several areas of research of human diseases viz. AIDS, Parkinson’s disease, hepatitis, dentistry, orthopaedic surgical techniques, cardiovascular surgeries, psychological disorders, toxicological studies, drug development, toxicological studies as well as vaccine development [4]. The discovery of vaccines and diagnostic modalities with the animal model does not only benefit humans but also enhances the lifespan of animals and prevents many zoonotic diseases, with the production of many vaccines and drugs like rabies, tetanus, parvo virus, feline leukemia, etc (Table 1).

**Table 1:** Significance and challenges of different animal models

Disease model/procedure	Animal model		References
	Significance	Challenges	
Ischemia and reperfusion injury of the spinal cord	Animal models are warranted	But, need several models are required (Pig, rabbit, mouse)	[9]
Cartilage defect repair with biomaterials	There are murine, ovine, leporine, caprine, porcine, canine, and equine models	In regards to cartilage thickness, joint biomechanics and ethical and licensing matters, caprine models are the best suited	[10]
Monoclonal antibodies for cancer treatment	Preclinical trials of monoclonal antibodies (mAbs) in animal models are required to reach the clinic	But, mAbs are less adapted to animal studies	[11]
Animal models to study of limb restoration	Cockroach: similar resemblance within the animal kingdom, cheap, least ethical regulations	Not ideal for the less resemblance with human	[12]
	Zebrafish: genome is well identified, vertebrate; grow very fast, high regenerative capacity, least ethical regulations	Not ideal for the less resemblance with human	[13, 14]
	Mouse: cheap, fast growth, well established genome, many species and transgenic strains, mammalian	Findings not trustworthy for human trials	[15–17]
	Rat: larger than mice, cheap, fast growth, well established genome, many species and transgenic strains, mammalian	Findings not trustworthy for human trials as well as maintenance cost is more than mice	[18–21]
	Dog: large in size, higher physical activity, cheaper than horse, mammalian, good for preclinical trial, results are trustworthy for human trials	More ethical constraints, more maturity period than rodents, expensive rearing cost	[22–26]
	Horse: larger mammal than dog, higher physical activity, trial result can easily be transferred to human	More ethical constraints, more maturity period, expensive rearing cost	[27–30]
Development of antibacterials	Efficacy and toxicity of antibacterials can be studied	But, animal model can’t predict human response to that component	[31]
Streptozotocin (STZ)—induced diabetes model	STZ produces clinical features in animals that resemble diabetes in humans	But, physiochemical properties and toxicities of STZ cause mortality to the animals	[32]

### Ethical matters on the use of animals

Animal research adheres to a few dimensions like government legislation, public opinion, moral stand, and search for appropriate alternatives for the research. Mahatma Gandhi opined that to judge the greatness and moral progress of a nation, one should judge the way its animals are being treated. Government legislation restricts the researchers and institutes from likely injury, pain, or suffering that may arise during animal research [33]. On the contrary, many modern countries ruled that before human administration, vaccine testing, lethal dose testing should be done on animals [34]. Social acceptance has also an influential role in animal experiments as it utilizes public money [33]. In their moral view, many people think that dog has more moral impact than pig, rat, fishes, mouse, etc.

Ethical issues on animal experimentation started in 1959, where the emphasis has been given on principles of 3Rs, reduction, refinement, and replacement of animal use [35]. According to this principle, minimum necessary numbers of animals are to be used for scientific experiments i.e. reduction. Pain or distress of the animals during experiments has to be minimized, i.e. refinement. Wherever applicable replacements of the animals are to be done with other non-animal alternatives, i.e. replacement. Though these principles are considered as the cornerstone of animal experimentations, but there are questions regarding the implementation of these regulations [36].

### Laboratory (small) and large animal models for human diseases

The importance of rat and mouse models has proved their outstanding importance in biomedical research. Besides, other mammalian and non-mammalian small domestic animals like the guinea pig, hamster, rabbit, ferrets, birds, amphibians, fishes, flies, worms have equal importance in terms of anatomical and physiological resemblance with humans. Large animal models also proved their uniqueness due to specific anatomical and physiological characteristics pertinent to those specific researches (Table 2).

**Table 2:** Biomedical significances and limitations of small animal models

Small animal models	Significances and limitations	References
Rats (*Rattus norvegicus domestica*) and Mice (*Mus musculus*) model	Easy breeding, handling, less rearing care, easily interchangeable between rats and mice. They are mostly inbred, so do not have genetic variations like a human, not a suitable model for inflammation study	[37–42]
Guinea pig (*Cavia porcellus)*	Mostly outbred, suitable for cholesterol metabolism, asthma model, feto-placental development and parturition, Alzheimer’s disease study, tuberculosis research, vaccine study. High phenotypic variations, Ebola research in guinea pig is limited due to the poor infectious potential of the virus	[43–63]
Hamster, especially golden hamster (*Mesocricetus auretus*)	Excellent for reproductive research due to the strict progesterone, but not oestrogen, short gestation period, unique an anatomical feature like loose subcutaneous space, important for micro-circulation studies, cancer model, infection model for leptospirosis, vaccine studies	[64–81]
Rabbit (*Oryctolagus cuniculus*)	Good model for surgically created osteoarthritis, wound healing model, drug study, asthma model, cholesterol model, cardiovascular disease model, Alzheimer’s disease model	[82–97]
Equids (*Equus*)	Important for the study of articular defects, orthopaedic models, tendinopathies, asthma model, reproductive models. But, more care expenses are required	[98–102]
Cattle (*Bos taurus*)	Important for study of female reproductive model, pregnancy related issues, tuberculosis models. But, more care expenses are required	[103–107]
Goat (*Capra hircus*)	Potential for orthopaedic studies, mechanical circulatory support devices, model for female to male XX sex reversal	[108–116]
Sheep (*Ovis aries*)	Easy to handle, easy sampling, physiological and anatomical nature are similar to humans, good for surgical model for bone and wound healing, asthma model, heart pathology, vaccine development, but, mostly inbred strains	[117–129]
Cat (*Felis catus*)	Important models for asthma, obesity, type-2 diabetes mellitus, HIV, cerebral palsy	[130–140]
Dog (*Canis familiaris*)	Narcolepsy, hemophilia B, or hereditary diseases, cancer, musculoskeletal research, etc	[141–150]
Pig (*Sus scrofa*)	Large litter size, more similar with human physiology, important for cardiovascular study, Alzheimer’s disease, Atherosclerosis, Type 2 diabetes mellitus, Breast cancer, etc	[151–156]

### Transgenic animal models in biomedical research

The gene rule and role in the biological system of human diseases has improved many folds with the introduction of the transgenic animal model in biomedical research within the last three decades. The early example of most unique biological research started, when structural gene coding for the human growth hormone (GH) was initiated into mice after fusion with the regulatory region of mouse metallothionein-I gene, as a result, transgenic mouse produced and showed excess GH production [157].

Linking of the genotype with disease phenotype has been expedited with the genome editing with the introduction of the CRISPR–Cas9 system by which disease-causing mutations are done in animal models [158]. Moreover, the production of transgenic animals has been radically changed by the introduction of the CRISPR–Cas9 system. Through the successful use of this model accurate human disease models in animals have been produced and possible therapies have been potentiated. Recapitulation of various disease-causing single nucleotide polymorphisms (SNPs) in animal models is achieved by the introduction of gRNA with the combination of Cas9 and donor template DNA [159], viz. mouse model has enormous importance in carrying human genetic traits, developmental similarities as well as disease translation [158, 160–162]. Zhang and Sharp labs at MIT/Broad Institute used CRISPR–Cas9 through AAV and lentivirus [163] both in vivo and ex vivo in neurons as well as endothelial cells of mice for the production of lung cancer model in mice where lung causing genes namely Kras, Tp53, and Lkb1 were mutated. On the other hand, an MIT-Harvard team [164] disrupted the tumor suppressor genes Pten and Tp53, and consequently liver cancer was produced in mice.

### Animal models in pharmaceutical drug development

In recent advancements, animal models are the most practical tools for pre-clinical drug screening before application into clinical trials. Animal models are considered as most important in vivo models in terms of basic pharmacokinetic parameters like drug efficiency, safety, toxicological studies, as these pre-clinical data are required before translating into humans. Toxicological tests are performed on a large number of animals like general toxicity, mutagenicity, carcinogenicity, and teratogenicity and to evaluate whether the drugs are irritant to eyes and skin. In most instances, both in vitro and in vivo models are corroborated before proceeding to medical trials. In vivo models are mostly conducted in mice, rats, and rabbits [2]. Certain stages are involved in pre-clinical trials with animal models: firstly, if the trial drug shows desirable efficacy then only further studies are carried out; secondly, if a drug in pre-clinical trials on animals proved to be safe, then it is administered in small human volunteer groups, at the same time, the animal trial will go on to evaluate the effect of the drug when administered for an extended period [8, 165]. Mostly, rodents are used for these trials as they have similar biological properties to humans and are easy to handle and rear in laboratories. In new regulations, it is mandatory to carry on the trials on non-rodents such as rabbits, dogs, cats, or primates simultaneously with rodents [166].

### Animal models in orthopedic research

There are many conditions involving bone pathologies such as osteomyelitis, osteosarcoma, osteoporosis, etc. Being a complex organ, the treatment of bone needs special care and extensive researches that involves specialized techniques as well as specific animal models for the studies of specific diseases. Herein, the animal models emphasize mostly related to fracture healing (critical size defect), osteoporosis, osteomyelitis, and osteosarcoma (Table 3).

**Table 3:** Different animal models in orthopaedic research

Animal model	Name of the procedure	Anaesthetic protocol	Procedure	Significance and limitations	References
Rat model	Critical size bone defect	Induction: 4% (vol/vol) isoflurane in oxygen for ~ 2 min. Maintenance of anesthesia with 2% (wt/vol) isoflurane. Administration of intraperitoneal (IP) injections of 0.05 mg/kg buprenorphine with 25 gauge needle for peri-operative analgesia and 5 ml/kg sterile normal saline with 18 gauge needle to account for fluid losses during surgery. Provides 30 min anesthesia	5 mm diameter of bilateral calvarial bone defect	The rat femur has more soft tissue coverage than other bones and the model has the potentiality to replicate the risk factors of non-union as humans. Haversian system is lacking, rotational stability is not achieved with only k-wire/intramedullary pins	[167–173]
Rabbit model	Critical size bone defect (Fig. 1a)	Intramuscular injection of Xylazine hydrochloride (5 mg/kg BW) and ketamine hydrochloride (50 mg/kg BW)	15 mm critical radial defect at distal diaphysis	Similar bone density with humans, though size and shape are different, as well as different in bone microstructure. Tibia and the less-weight carrying bones are more used	[168, 172–180]
Goat and sheep	Segmental bone defect	Intramuscular injection of Xylazine hydrochloride @ 0.1–0.2 mg/kg BW	3 cm defect in femur, tibia, radius, and metatarsus	Similar body weight and bone size like humans. Plexiform bone is predominant; Haversian remodeling can be seen in the later stage of the life cycle. Different bone metabolism as compared to monogastric animals	[171, 181–186]
Rabbit model	Osteomyelitis (Fig. 1d)	Intramuscular injection of Xylazine hydrochloride (5 mg/kg BW) and ketamine hydrochloride (50 mg/kg BW)	A needle is to be introduced into the proximal femur medullary cavity, 1 mL of bone marrow is to be removed and replaced with 0.1 mL 5% sodium morrhuate and 0.1 mL of *Staphylococcus* suspension (Kanin strain, 3 × 10^6^ cfu/mL). The opening point is to be sealed with bone wax	Rabbit bones are ideal for plate and screw fixation and the medullary canal of the tibia and femur are capable to accommodate internal implants. But, a higher dose of inoculation 10^3^–10^8^ CFU is required for successful infection	[187–193]
Rat model	Osteomyelitis	Induction: 4% (vol/vol) isoflurane in oxygen for ~ 2 min. Maintenance of anesthesia with 2% (wt/vol) isoflurane. Administration of intraperitoneal injections of 0.05 mg/kg buprenorphine with 25 gauge needle for perioperative analgesia and 5 ml/kg sterile normal saline with 18 gauge needle to account for fluid losses during surgery. Provides 30 min anesthesia	K wire is to be inserted into the medullary cavity of tibia and then 5% sodium morrhuate injection followed by a *S. aureus* suspension (10^2^ cfu/10 μL) is to be injected into the tibial metaphysic. To prevent bacterial leakage fibrin glue and sealant is to be used	Bones in the rat are suitable for a different pattern of fracture and intramedullary implants. But rats require 10^3^–10^6^ CFU inoculation dose	[191, 193, 194]
Goat model	Osteomyelitis	Intramuscular injection of Xylazine hydrochloride @ 0.1–0.2 mg/kg BW	3-mm drill hole is to be made in distal tibia and injection of 1 mL 5% sodium morrhuate, afterwards an injection 10 min later with *S. aureus* (7.05 × 10^4^ cfu). To prevent bacterial leakage fibrin glue and sealant is to be used	They are larger than other species under study hence implants and prostheses that are used in humans can be used in goats successfully. But they are expensive as well as the raring cost is more. Inoculation dose is 10^3^–10^5^ CFU in goat models	[191, 193, 195]
Rabbit model	Osteoporosis	Intramuscular injection of Xylazine hydrochloride (5 mg/kg BW) and ketamine hydrochloride (50 mg/kg BW)	Bilateral ovariectomy afterwards IM injection of 1 mg/kg BW/day of methylprednisolone for 4 weeks	They achieve early skeletal maturity than other mammals	[196–200]
Sheep model	Osteoporosis	General anesthesia with intramuscular injection of Xylazine hydrochloride @ 0.1–0.2 mg/kg BW	Bilateral ovariectomy, low calcium diet, weekly IM administration of dexamethasone for 6 weeks	They are docile, easy to handle, and house. Bone size similar to human. But, as they are ruminant, hence, oral drug administration does not yield the desired result. Surgical intervention is required to create an abomasal fistula	[201–204]
Mouse model	Osteosarcoma	Isoflurane/oxygen-based anesthesia for induction then maintenance by IM administration of Xylazine @10 mg/kg BW and ketamine @100 mg/kg BW	After the preparation of osteosarcoma cells as described by Uluçkan et al., a 0.5 cm skin incision is made just below the knee to expose tibial tuberosity, then cells are injected into the medullary cavity with 26–28 G syringe and skin is sutured	Cheap availability, easy to handle, genetic similarity with humans. Hence, become important for oncological research	[205–207]

### Animal models in diabetic and burn wound healing

Type 2 diabetes and associated foot ulcer have turned into an epidemic worldwide in recent years causing severe socio-economic trouble to the patients as well as the health care system of the nation as a whole [208]. Various researches depicted that chance of developing an ulcer in diabetic patients varies between 15–25% [209, 210] and the chance of recurrence is about 20–58% among the patients within a year after recovery [211]. Hence, many researchers studied different materials or drugs to treat diabetic wounds. Similarly, burn wounds occur due to exposure to flames, hot surfaces, liquids, chemicals, or even cold exposure [212]. Though with the recent modalities like skin grafting prognosis has improved however, the mortality rate is high [213–215].

### Diabetic wound rat model

**Fig. 1:**
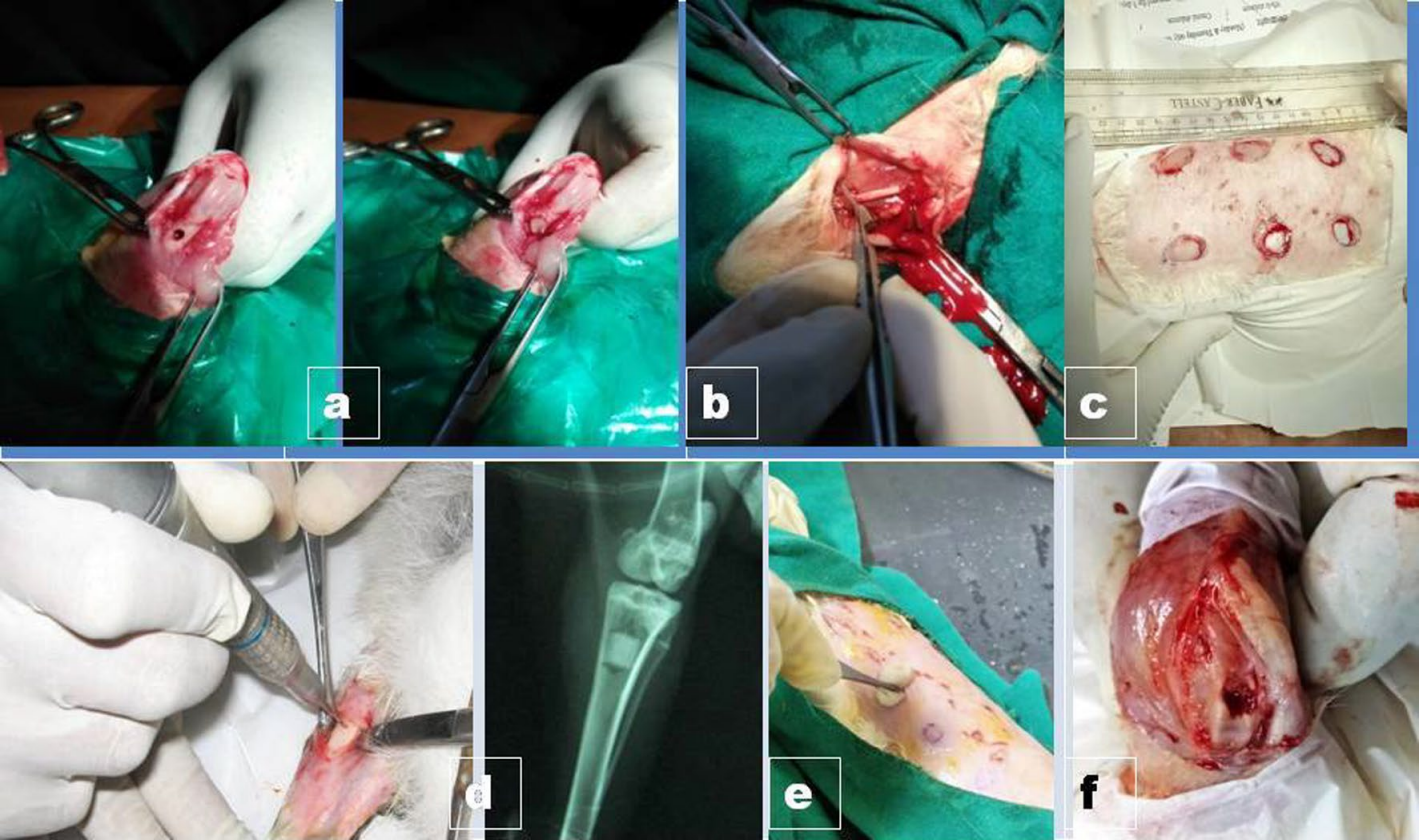
**a**. Bone defect model and implantation of implant **b**. Vascular graft mode **c**. Diabetic wound model **d**. Osteomyelitis model development **e**. Creation of burn wound model **f**. Cartilage graft model—All in rabbit

For developing this model, clinically healthy male Wistar rats (150 ~ 250 g body weight) are used. To induce hyperglycemia, injection nicotinamide (NAD)@ 150 mg/kg BW intraperitoneally, after 15 min injection Streptozotocin (STZ) @ 65 mg/kg BW intraperitoneally [216] are to be injected. The same procedure has to be repeated after 24 h. Blood is to be collected from the tail after 72 h to check hyperglycemia. Rats having high blood glucose levels (≥ 10 mmol/L) are considered to be diabetic [217]. For wound creation, rats are to be anesthetized with a combination of xylazine @10 mg/kg (intramuscular injection) and ketamine @90 mg/kg (intramuscular injection) [218]. After marking the dorsal back area with methylene blue, the site is to be prepared aseptically after shaving [219]. Full-thickness wound creation is to be done with a sterile 6 mm biopsy punch measuring 6 mm diameter and 2 mm depth and left open [218] (Fig. 1c).

### Burn wound models

Because of the severity and types of cause, the management of burn injuries poses a significant challenge to plastic surgeons in humans. In general, primary and secondary burn wounds heal by the primary healing process, but, third-degree burn injuries with the destruction of all the skin layers are resistant to the normal healing process and necessitate the added surgical procedures, such as skin grafting, and the relevance of advanced wound dressing [220]. Several researchers used the albino Winstar male rats (*Rattus norvegicus*) model weighing 250 ± 50 g for the study of burn wounds. Anesthesia was achieved with intramuscular administration of atropine sulfate (0.04 mg/kg BW) and after 10 min a combination of 10% ketamine (90 mg/kg) and 2% xylazine (10 mg/kg) intramuscularly produced adequate anesthesia [221]. After aseptic preparation of the dorsal back area, thermal injury has to be made with a 10 mm aluminium rod previously heated with 100 °C boiling water. The aluminium rod has to be kept in situ for 15 s. Immediately after the procedure analgesic is to be provided and to be continued for at least 3 days [222–224]. A hot air blower has been used to produce a 6% third-degree burn injury in a mouse model [225]. In pig, a partial-thickness burn model in the skin was produced by placing a glass bottle having heated water at 92 °C for 14 s [226] In other studies, a homemade heating device was placed over the skin for 35 s to create burn wound [227]. In rabbits, it was demonstrated to use a dry-heated brass rod for 10 and 20 s at 90 °C to create a deep partial-thickness burn wound in the ear [228]. In mice, a full-thickness burn was created under 3–5% isoflurane anesthesia and intraperitoneal caprofen 5 mg/kg as analgesia. Here, a 4 cm^2^ brass rod attached to a temperature probe was first heated to 260 °C and then cool to 230 °C and finally placed on the dorsum skin for 9 s [229] (Fig. 1e).

### Animal models in cartilage repair

Animal models have enormous importance in the study of cartilage repair. Though in vitro models have been reported, it could not replace the necessity of using animal models prior to clinical implementation [230–236] (Table 4).

**Table 4:** Different animal models for cartilage rejuvenation or repair

Animal model	Anesthesia	Procedure	Significance and limitations	References
Rabbit	Intramuscular injection of Xylazine hydrochloride (5 mg/kg BW) and ketamine hydrochloride (50 mg/kg BW)	3 mm diameter critical size defect at shoulder or knee, depth 0.2–0.5 mm at the chondral or osteochondral site (Fig. 1f)	Low cost, easy to handle, and house, but different from humans in respect of biomechanics due to their different hopping and walking pattern	[10, 82, 232, 237–239]
Sheep/Goat	General anesthesia with intramuscular injection of Xylazine hydrochloride @ 0.1–0.2 mg/kg BW	Knee joint surgically exposed and 6–7 mm circular critical defect is to be created with 0.4–1.5 mm depth at chondral/osteochondral site	Easy to rare, handle and have close anatomical similarity with humans but knee contact areas are different, hence this must be considered	[10, 232, 240–245]
Dog	General anesthesia using preanesthetic atropine sulphate @0.04 mg/kg BW SC, after 10 min xylazine 1–2 mg/kg BW IM. Maintenance by ketamine @5–10 mg/ kg BW IV and diazepam 0.5 mg/ kg BW slow IV	Surgically created 4 mm diameter circular critical size defect of 0.95–1.3 mm depth at the chondral/osteochondral site of Knee, shoulder, elbow, hip or ankle joint	They are a good model for cartilage repair as they can be trained for treadmill walking, swimming, etc. But, disadvantages are there. Firstly, ethical issues in several countries, moreover canine cartilage is thinner compared to human and anatomical difference exists in the knee joint	[10, 230, 244, 246–248]

### Animal models in vascular grafting

With the increase of cardiovascular complications, there is a need for surgical intervention using vascular grafts. Vascular grafting and cardiac valve repair have become important issues to the clinicians for the replacement of damaged vessels [249, 250], hence there is an increased demand for tissue-engineered blood vessel substitute [250, 251]. The main prosthetic options are synthetic grafts such as polytetrafluoroethylene, polyethylene terephthalate, and polyurethane [252], and autologous conduits. Although these types of synthetic grafts provide reasonable outcomes in large-diameter vascular applications, long-term patency is questionable as compared to autologous conduits in small-diameter (< 6 mm) applications due to their inclination to various complications [253]. Despite the superior outcome of autologous grafts, it has some disadvantages such as limited availability and prior use. Moreover, the determination of a suitable animal model needs considerations of various factors. The factors for the selection of animal species depend on diameter and length of conduits, period of implantation, anastomotic site, price, accessibility, reaction to anesthesia and surgery, and flow of blood at sites of graft implantation. Animal applications of these tissue-engineered vessels are, therefore, an utmost necessity as pre-clinical studies before use in humans (Fig. 1b, Table 5).

**Table 5:** In vivo animal studies of different vascular grafts

Animal species	Type of graft	Graft diameter (mm)	Graft patency rate	In vivo study model	References
Ovine	EC-seeded xenogenic porcine decellularized carotid artery	5		Common carotid artery/external jugular vein arteriovenous shunt	[254]
Canine	PCL + VEGF	2	100% in 4 weeks	Femoral artery	[255]
Canine	P(LLA-CL) + Autologus, EC preendothelialization	4	88.9% in 24 weeks	Femoral artery	[256]
Canine	P(LLA-CL)	4	75% in 3 months	Femoral artery	[257]
Ovine	Decellularized graft derived from fibrin gel and ovine dermal fibroblasts	4	100% in 168 days	Carotid artery	[258]
Ovine	Heparin and VEGF-treated xenogenic porcine dSIS	5	92% in 90 days	Carotid artery	[259]
Mouse	PCL	0.5	53% in 28 days	Carotid artery	[260]
Rabbit	P(LLA-CL) + Collagen + Elastin + VEGF	4	86% in 3 weeks	Infrarenal aorta	[261]
Ovine	PCL electrospun + PLCL sponge	5	100% in 8 weeks	Carotid artery	[262]
Ovine	PHBV/PCL-GF	4	50% in 1 year	Carotid artery	[263]

### Animal models in disc degeneration

Intervertebral disc degeneration (IVDD) and herniation manifested as lower back pain cause a massive socio-economic burden to the patient and society as a whole [264–267]. But there is a lack of treatment modalities to cure mildly to moderate degeneration as well as complications associated with surgical interventions associated with the advanced stage; hence, researchers are enormously trying to reinforce regenerative strategies and to lower the suffering by controlling the pain with the injection of stem cells, growth factors hydrogels for replacement of the disc [268]. Diverse animal models have been reported as a pre-clinical trial to translate the procedure in humans (Table 6).

**Table 6:** Different animal models for the study of IVDD

Animal model	Anaesthesia	Procedure	Significance and limitations	References
Goat	Ketamine (11–33 mg/kg BW) and midazolam (0.5–1.5 mg/kg BW), intravenously followed by maintenance with an isoflurane-oxygen combination	Following the aseptic technique, the lumbar intervertebral discs were opened via left lateral retroperitoneal, transpsoatic approach. A titanium Kirschner wire was positioned in the L1 or L2 vertebral body to facilitate marking of vertebral levels on radiographs	Weight range, disc height, size, and shape are similar to humans. They can withstand the stress of anaesthesia and surgery well. But, goat torse has a different anatomical structure in comparison to a human	[268–272]
Rabbit	Intramuscular injection of Xylazine hydrochloride (5 mg/kg BW) and ketamine hydrochloride (50 mg/kg BW)	After positioning the rabbit in lateral decubitus position a 20 degrees inclination was produced. IVD was exposed with a posterolateral retroperitoneal approach. After dissecting the skin, subcutaneous tissue, and muscle, the left anterolateral aspect of L1–L5 was exposed. Then, one IVD is punctured between L1–L5 with the help of a 16-gauge needle to a depth of 5 mm in the left anterolateral annulus fibrosus in the annular stab method	Similar to human disc degeneration in biochemical and histological aspects. But, the method causes rapid narrowing of the disc space and disc height as well as rapid herniation of nucleus pulposus	[273–280]

## Conclusions

The importance of animal models is unquestionable in terms of in vivo study for the implementation of any biomedical research to humans. It serves not only the human race but also well being of veterinary patients. Animal models have not only important roles in drug development, toxicity studies, pharmacokinetic studies of a drug, but also the pre-clinical study of medical and tissue engineering devices that are intended to be used in humans. Laboratory animal models are more cost-effective and agreeable to high throughput testing as compared to large animal models. Yet, to obtain preclinical data and to ascertain the clinical potential of vascular graft as well as orthopedic bone plates and implants, large animal models that mimic human anatomy and physiology are to be developed. Whatever may be the modes of using animal models for biomedical researches, it should abide by the principles of 3Rs, i.e., reduction, refinement, and replacement of animals.

## Data Availability

The data in the present manuscript were collected by searching of literatures as well as involving authors own materials.

## Abbreviations

BW: Body weight
Cfu: Colony forming unit
ESC: Embryonic stem cell
IVDD: Intervertebral disc degeneration
PCL: Polycaprolactone
STZ: Streptozotocin
VEGF: Vascular endothelial growth factor
